# Development of alcohol control law, Sao Tome and Principe

**DOI:** 10.2471/BLT.22.288590

**Published:** 2022-09-02

**Authors:** Zila M Sanchez, Arlindo Carvalho, Celmira Sacramento, Filomena D’Alva, Iazalde Rita, Sebastiao Pinheiro, Elisabete M Soares de Barros, Vilfrido Santana Gil, Me-Chinho Costa Alegre, Anne Ancia, Maristela G Monteiro, Chidinma A Opoko, Juan E Tello

**Affiliations:** a Department of Preventive Medicine, Federal University of Sao Paulo, Sao Paulo, Brazil.; b Sao Tome National Assembly of Deputies, Sao Tome, Sao Tome and Principe.; c Ministry of Health of Sao Tome and Principe, Sao Tome, Sao Tome and Principe.; d World Health Organization Country Office, Sao Tome, Sao Tome and Principe.; e Department of Noncommunicable Diseases and Mental Health, Pan American Health Organization, Washington, DC, United States of America.; f Cluster of Universal Health Coverage and Healthier Population, World Health Organization Regional Office for Africa, Brazzaville, Congo.; g Department of Health Promotion, World Health Organization, Avenue Appia 20, 1211 Geneva 27, Switzerland.

## Abstract

The World Health Organization (WHO) African Region is struggling with increasing harm associated with alcohol consumption. Legislators of Sao Tome and Principe, concerned about this harm and the high prevalence of alcohol use disorders, designed a comprehensive alcohol control bill to tackle this situation. Input into the design of the bill was obtained through interviews involving many stakeholders. The process had five phases: (i) scoping the problem to understand the social burden of the harm caused by alcohol consumption; (ii) updating the evidence on alcohol policies and identifying areas for legislative interventions; (iii) drafting the bill; (iv) aligning the legislative framework of the bill; and (v) initiating the parliamentary procedure. The new bill scored 92/100 using a standardized alcohol control policy scale. The bill covers all domains of WHO’s 2010 global strategy to reduce the harmful use of alcohol, and includes the three most cost-effective interventions for reducing alcohol consumption: increased excise taxes on alcohol; bans or comprehensive restrictions on exposure to alcohol advertising; and restrictions on the availability of retailed alcohol through reduced hours of sale. The National Assembly plenary session upheld the bill, which is now under evaluation of the specialized First Commission on Political, Legal, Constitutional and Ethical Affairs. Approval of the bill requires the final voting once it is back with the National Assembly and its promulgation by the President. Drafting an alcohol control bill which is country-led, inclusive, evidence-based and free of interference by the alcohol industry helps prioritize public health objectives over other interests.

## Résumé

La Région africaine de l'Organisation mondiale de la Santé (OMS) fait face à une hausse des dégâts causés par l'alcool. Préoccupés par la situation et par la forte prévalence des troubles liés à cette consommation, les législateurs de Sao Tomé-et-Principe ont élaboré un projet de loi détaillé afin d'y remédier. À l'origine de sa conception, plusieurs entretiens avec différentes parties prenantes. Le processus s'est divisé en cinq phases: (i) définir l'étendue du problème pour évaluer le fardeau que les dégâts provoqués par l'alcool font peser sur la société; (ii) actualiser les données probantes relatives aux politiques en matière d'alcool et identifier les domaines nécessitant une intervention législative; (iii) rédiger le projet de loi; (iv) aligner le cadre législatif du projet de loi; et enfin, (v) initier la procédure parlementaire. Le nouveau projet de loi a obtenu un score de 92/100 sur une échelle d'évaluation standard des mesures de lutte contre l'alcool. Il couvre tous les thèmes repris dans la Stratégie mondiale de l'OMS visant à réduire l'usage nocif de l'alcool, publiée en 2010. Il prévoit également les trois interventions les plus rentables en termes de diminution de la consommation d'alcool: l'augmentation des taxes d'accise sur l'alcool; l'interdiction ou l'instauration de conditions strictes en matière d'exposition à la publicité pour l'alcool; et une disponibilité restreinte des boissons alcoolisées dans le commerce en limitant les heures de vente. L'Assemblée nationale a soutenu le projet de loi en séance plénière. Il est désormais en cours d'évaluation au sein de la première Commission spécialisée en affaires politiques, juridiques, constitutionnelles et éthiques. Pour être adopté, le projet de loi doit revenir à l'Assemblée nationale pour un vote final, puis être promulgué par le président. Une loi globale de lutte contre l'alcool, élaborée par le pays lui-même, fondée sur des faits et n'ayant subi aucune ingérence de la part de l'industrie de l'alcool contribue à faire passer les objectifs de santé publique avant d'autres intérêts.

## Resumen

La Región de África de la Organización Mundial de la Salud (OMS) está tratando de hacer frente a los crecientes daños derivados del consumo de alcohol. Los legisladores de Santo Tomé y Príncipe, preocupados por estos daños y por la alta prevalencia de los trastornos por consumo de alcohol, diseñaron un proyecto de ley integral para el control del alcohol con el fin de solucionar esta situación. Las contribuciones al diseño del proyecto de ley se obtuvieron a través de entrevistas en las que participaron muchas partes interesadas. El proceso constó de cinco fases: (i) la delimitación del problema para comprender la carga social de los daños causados por el consumo de alcohol; (ii) la actualización de los datos sobre las políticas de alcohol y la identificación de las áreas de intervención legislativa; (iii) la redacción del proyecto de ley; (iv) la adaptación del marco legislativo del proyecto de ley; y (v) el inicio del procedimiento parlamentario. El nuevo proyecto de ley obtuvo una puntuación de 92/100 según una escala estandarizada de políticas para el control del alcohol. El proyecto de ley abarca todos los ámbitos de la estrategia mundial que la OMS puso en marcha en 2010 para reducir el uso nocivo del alcohol, e incluye las tres intervenciones más rentables para reducir el consumo de alcohol: el aumento de los impuestos especiales sobre el alcohol; la prohibición o la restricción general de la exposición a la publicidad del alcohol; y la restricción de la disponibilidad del alcohol al por menor mediante la reducción del horario de venta. La sesión plenaria de la Asamblea Nacional respaldó el proyecto de ley, que ahora está bajo evaluación de la Comisión Primera especializada en Asuntos Políticos, Jurídicos, Constitucionales y Éticos. La aprobación del proyecto de ley requiere la votación final una vez que regrese a la Asamblea Nacional y que el Presidente lo promulgue. La elaboración de un proyecto de ley para el control del alcohol que sea liderado por el país, inclusivo, basado en la evidencia y libre de interferencias por parte de la industria del alcohol ayuda a priorizar los objetivos de salud pública sobre otros intereses.

## ملخص

تعاني المنطقة الأفريقية التابعة لمنظمة الصحة العالمية (WHO) من الضرر المتزايد المرتبط باستهلاك الكحول. إن المشرعين المعنيين بهذا الضرر وبانتشار مشكلات تعاطي الكحول، في سان تومي وبرينسيبي، قاموا بوضع مشروع قانون شامل لمكافحة الكحول بهدف معالجة هذا الوضع. تم الحصول على معطيات تصميم القانون من خلال المقابلات الشخصية مع العديد من أصحاب المصلحة. تضمنت العملية خمس مراحل: (1) تحديد نطاق المشكلة لفهم العبء الاجتماعي للضرر الناجم عن استهلاك الكحول؛ و(2) تحديث الأدلة المتعلقة بسياسات الكحول وتحديد مجالات التدخل التشريعي؛ و(3) صياغة مشروع القانون؛ و(4) تنسيق الإطار التشريعي لمشروع القانون؛ و(5) بدء الإجراءات البرلمانية. سجل مشروع القانون الجديد 92/100 باستخدام مقياس موحد لسياسة مكافحة الكحول. يغطي مشروع القانون جميع نطاقات الاستراتيجية العالمية لمنظمة الصحة العالمية لعام 2010، للحد من الاستخدام الضار للكحول، ويتضمن التدخلات الثلاثة الأكثر فعالية من حيث التكلفة للحد من استهلاك الكحول: زيادة الضرائب المفروضة على الكحول؛ عمليات الحظر أو القيود الشاملة على التعرض لإعلانات الكحول؛ والقيود المفروضة على توافر المشروبات الكحولية بالتجزئة من خلال تخفيض ساعات البيع. وأيدت الجلسة العمومية للجمعية الوطنية مشروع القانون، والذي يخضع الآن لتقييم اللجنة الأولى المتخصصة للشؤون السياسية والقانونية والدستورية والأخلاقية. تتطلب الموافقة على مشروع القانون، التصويت النهائي بمجرد عودته إلى الجمعية الوطنية وإصداره من جانب الرئيس. إن صياغة مشروع قانون لمكافحة الكحول تحت قيادة الدولة، يتمتع بالشمولية، والاعتماد على الأدلة، ودون التدخل من جانب صناعة الكحول، يساعد في منح الأولوية لأهداف الصحة العامة على المصالح الأخرى.

## 摘要

世卫组织 (WHO) 非洲区域办事处当前面临日益严重的饮酒危害问题。圣多美和普林西比的立法者担心这种危害加剧以及酒精使用障碍发病率过高，制定了一项全面的酒精控制法案来解决这一问题。在法案设计阶段，通过采访收集了许多利益相关者提供的建议。该过程分为五个阶段：(i) 界定问题范围以了解饮酒的危害及其造成的社会负担；(ii) 更新有关酒精政策的证据，并确定立法干预的领域；(iii) 起草法案；(iv) 调整法案的立法框架；以及 (v) 启动议会议事程序。根据标准化酒精控制政策量表，新法案得分为 92/100。新法案涵盖了世卫组织于 2010 年公布的减少有害使用酒精全球战略的所有领域，并列出了三个最具成本效益的饮酒量控制干预措施：增加酒精消费税；禁止或全面限制酒精类广告的宣传；通过缩短销售时间限制零售酒的供应。国民议会全体会议支持该法案，且第一政治、法律、宪法和道德事务特设委员会目前正在评估该法案。该法案需要在送回国民议会进行最终投票并由总统签署后方可生效。起草由国家主导、具有包容性、以证据为基础且不受酒精行业干预的酒精控制法案有助于优先实现公共卫生目标而不是考虑其他利益。

## Резюме

Африканский регион Всемирной организации здравоохранения (ВОЗ) борется с растущим вредом, связанным с употреблением алкоголя. Обеспокоенные этим вредом и высокой распространенностью расстройств, связанных с употреблением алкоголя, законодательные органы Сан-Томе и Принсипи разработали всеобъемлющий законопроект по борьбе с алкоголем для решения данной ситуации. Свой вклад в разработку законопроекта внесли многие заинтересованные стороны путем участия в опросах. Процесс состоял из пяти этапов: (i) определение масштаба проблемы для понимания социального бремени вреда, причиняемого употреблением алкоголя; (ii) обновление данных о политике в отношении употребления алкоголя и определение областей для законодательных мер; (iii) составление законопроекта; (iv) согласование законодательной основы законопроекта; (v) инициирование парламентской процедуры. Новый законопроект набрал 92 балла из 100 по стандартной шкале для оценки эффективности политики в отношении алкоголя. Законопроект охватывает все области глобальной стратегии ВОЗ сокращения вредного употребления алкоголя от 2010 г. и включает три наиболее эффективных с точки зрения затрат мероприятия по сокращению потребления алкоголя: повышение акцизов на алкоголь, запреты или всеобъемлющие ограничения воздействия рекламы алкоголя и ограничения доступности алкоголя в розничной сети за счет сокращения часов продажи. Пленарное заседание Национальной ассамблеи поддержало законопроект, который в настоящее время находится на рассмотрении специализированной Первой комиссии по политическим, правовым, конституционным и этическим вопросам. Утверждение законопроекта требует окончательного голосования после его возвращения в Национальную ассамблею и обнародования президентом. Разработка законопроекта по борьбе с алкоголем, который является инициативой страны, инклюзивен, основан на фактических данных и свободен от вмешательства алкогольной промышленности, помогает считать цели общественного здравоохранения приоритетными по отношению к другим интересам.

## Introduction

For several decades, alcohol consumption has been one of the leading factors for preventable deaths and the burden of diseases globally,^1^ causing 3 million deaths annually.^2^ Higher alcohol-related mortality and morbidity rates are found in lower-income and more vulnerable populations.^3^ In Africa, per capita alcohol consumption has increased. In 2012, consumption was 14.5 L of pure alcohol per drinker per year; in 2016, consumption increased to 18.4 L. In both years, drinkers in Africa consumed 3 L more than the world mean.^2^

In Sao Tome and Principe, 76% (1828/2418) of those aged 18 years and older are current drinkers, and one in four men drinks daily.^4^ An estimated 60% of men who drank were engaged in heavy episodic drinking, that is, consuming at least 60 g of pure ethanol per man at least once in the past month, which is 2.5 times higher than the prevalence for women.^2^ Per capita alcohol consumption in the population is 6.8 L per year, which is higher than the average of the World Health Organization (WHO) African Region (6.3 L). Among drinkers, per capita alcohol consumption is 20.1 L, also above the average in Africa (18.4 L). Alcohol use disorders in the Sao Tome and Principe population are almost twice that in the African Region (6.4% versus 3.7%).^2^ In addition, Sao Tome and Principe has an increasing prevalence of crime, cases of domestic violence, sexual abuse of minors, road traffic incidents and absenteeism at work.^5^

This paper describes the development of the new Sao Tome and Principe national alcohol control bill and the tools and processes used.

## Alcohol policy scoring

In 2010, the *Global strategy to reduce the harmful use of alcohol*,^6^ adopted by the World Health Assembly, proposed effective policies and interventions for reducing alcohol harm, grouped into 10 policy domains. In the WHO African Region, a regional and aligned strategy was approved in September 2010.^7^

These international commitments were strengthened with the adoption of the *Global action plan for the prevention and control of noncommunicable diseases 2013–2020*^8^ and its monitoring framework,^9^ as well as with the adoption of the sustainable development goals, specifically Target 3.5 – Strengthen the prevention and treatment of substance abuse, including narcotic drug abuse and harmful use of alcohol.^10^ Nonetheless, progress in implementing effective alcohol control policies has been minimal and even more limited in low- and middle-income countries.^11^ To address this issue, the Seventy-fifth World Health Assembly in 2022 endorsed the 2022–2030 global action plan to effectively implement the global strategy to reduce the harmful use of alcohol as a public health priority.^12^^,^^13^

In 2017, the WHO Regional Office for Europe developed the Alcohol Policy Scale to assess countries’ progress in the 10 policy domains of the 2010 global strategy and identify policy gaps.^14^ In 2018, the Pan American Health Organization adapted this tool to assess progress in countries of the Region of the Americas.^15^ Similar tools have been developed in line with the 2010 global strategy that focus on high-impact interventions, such as the alcohol environment protocol^16^ and the protocol of the Organisation for Economic Co-operation and Development.^17^

This tool was used to assess Brazil’s alcohol control interventions which allowed civil society to understand the gaps and discuss national priorities with legislators.^18^

## Need for alcohol policies

Although most legislative reforms entail lengthy processes, the length of the process could be even longer when alcohol is involved, mainly because alcohol is often mistakenly seen as a harmless beverage or is linked to social and cultural heritage.^19^ Other obstacles faced during the writing of an alcohol control bill include: the vested interests and interference of the alcohol industry in all phases of the legislative discussion;^20^ resistance to increasing prices in low-income countries;^21^ and the need to control traditional unlicensed production and sale.^22^

Defiance and inertia in discussing alcohol control policies are evident 12 years after the Africa regional strategy provided guidance on priority interventions to be implemented in the context of the economic, social and cultural diversity of the WHO African Region.^7^

Economic development, the substantial investment of alcohol companies and the lack of awareness of policy-makers and the public about alcohol-related risks and harm expose the African Region to problems related to alcohol consumption and reluctance to discuss and implement public policies that would successfully protect the population.^22^

In this regional scenario, the island of Sao Tome and Principe, an African country near the Equator, began drafting a national alcohol bill. The initiative came from legislators concerned about trends in alcohol consumption and the associated harm in the country. The initiative was so innovative that we consider that sharing the process is both a guide and a stimulus for other African countries to start their own action on alcohol consumption. Only 30% (14/47) of countries in the WHO African Region have a written national alcohol policy, which suggests an urgent need for action.^2^

The Sao Tome and Principe national alcohol bill aims to protect the population’s health by integrating measures to regulate the market so as to reduce the harm within society and to treat individuals with alcohol use disorders, rather than forbidding the sale or consumption of alcoholic beverages.

## Development of the bill

The process applied a naturalistic inquiry^23^ and comprised five phases: (i) scoping the dimension of the problem to understand the social burden and harm of alcohol consumption; (ii) updating the evidence and identifying areas for legislative interventions, including political feasibility; (iii) collectively drafting the bill; (iv) aligning the legislative framework of the bill; and (v) initiating the parliamentary procedure.

### Scoping the problem

The Sao Tome and Principe Fifth Specialized Permanent Commission of the National Assembly of Deputies comprises nine deputies representing three political parties. The commission oversees national legislation on gender, family, social cohesion, youth, sports and media. The commission embarked on this process to address the increasing harm related to alcohol. From 2019 to 2021, the commission conducted eight informal conversational interviews with government officials and two fieldworks to interact with civil society in Roça-Lemba and Principe. These interviews included meetings with: the Minister of Youth, Sports and Entrepreneurship; the Minister of Justice, Public Administration and Human Rights; the Minister of Education and Higher Education; the Minister of Health; district council mayors of Água Grande, Cantagalo, Caué, Lobata, Lembá and Mé-Zóchi; and the Mayor of the Regional Government of Principe. In addition, the police, youth associations, community leaders, associations of taxi drivers, motorcyclists and women informal traders (*palaiês*), among others, were also consulted.

The consultative process allowed the commission to define critical topics, identify resistances across communities, and build the case for and justify the investment in the development of the bill. As a result, the commission gained insights into the scope of social and economic problems related to alcohol consumption and the readiness of civil society and officials for legislative intervention. The interviews were transcribed verbatim and analysed through a framework that generates timely information for stakeholders by using qualitative interviews to answer four domains for the decision-making policy process:^24^ contextual (form and nature of the problem); diagnostic (reasons for, or causes of, what was found in the contextual domain); evaluative (appraisal of the effectiveness of the interventions and strategies that exist); and strategic (definition of new theories, policies, plans or actions).

The ministers of education, youth and justice who participated in the informal conversational interviews confirmed support for a comprehensive national alcohol control law and noted specific interventions needed. These interventions included: increasing import taxes; prohibiting sales to minors and students; investing in prevention and life-skills training programmes; enforcing the inspection of alcohol sales; prohibiting consumption of alcohol in workplaces; and raising consumer awareness of the effects of alcohol consumption.

The mayors of the district councils brought up concerns about the need to strengthen control of the production and distribution of alcoholic beverages, especially *cacharamba* and *vinho de palma* (local alcoholic beverages), and access to alcohol. Importation was mentioned as a crucial factor to be regulated since a large proportion of the alcohol available is imported. All districts considered the problem of alcohol intoxication and the associated violence a particularly important issue.

Civil society also confirmed its support for a comprehensive bill given the increased violence associated with alcohol consumption, including sexual violence. The need to establish inspection mechanisms, address the production of alcoholic beverages and provide financial support for those families for whom alcohol production is the main source of household income was raised, as was the need for upstream interventions such as the provision of drinking water, classrooms and leisure space.

The financial dependence of some families on the production and sale of traditional alcoholic beverages, such as *cacharamba* and *vinho de palma*, is a peculiarity of Sao Tome and Principe and other African countries. This situation limited the scope of the bill. A bill with more severe restrictions on domestic producers would have greatly affected the subsistence of many families and may have increased illicit alcohol trading. Consideration for these cultural features was possible because of the engagement with civil society and district councils. However, there is evidence of high toxicity in homemade beverages in some African countries due to mycotoxin contamination.^25^

During the informal interviews, it was clear that stakeholders are not always aware of their role and responsibilities in alcohol control. In fact, the health sector has a convening role in many proposed interventions but other sectors are expected to play their part in implementation and enforcement. The new national alcohol bill could have defined actors’ roles and responsibilities.

### Identifying intervention areas

The bill aimed to cover the production, import, advertising, availability, marketing and consumption of alcoholic beverages to increase its effectiveness.

The commission requested WHO’s support to ensure the bill’s alignment with the 2010 global strategy to reduce the harmful use of alcohol and incorporate the most recent scientific evidence.

Two WHO experts supported the commission: a senior expert on alcohol policy and a national lawyer experienced in writing legislation. Fluency in Portuguese, the local language, was crucial to debating each aspect of the bill with legislators.

Cabo Verde’s, Brazil’s and Portugal’s national policies and laws on alcohol control were reviewed. The Cabo Verde alcohol control law was used as an initial reference law. This law’s adherence to the 2010 global strategy was reviewed using the alcohol policy scoring tool.^15^^,^^18^ The commission concluded that the Sao Tome and Principe national alcohol bill needed to expand in several domains not covered by the reference law and to respond to the problems identified during the informal interviews and fieldwork. Brazil’s and Portugal’s laws helped to fill those gaps.

### Drafting the bill

In August 2021, the Fifth Commission drafting the bill convened a 3-day retreat. Participants were undisturbed during the discussions. The participants included the nine deputies of the commission, a representative of the health ministry, a national lawyer, a WHO delegate and a senior expert on alcohol policy. No representative of the alcohol industry was included in this retreat or in any phase of the process. Participants identified gaps in national laws and proposed improvements for the new national alcohol bill using the alcohol policy scoring tool, which had been translated into Portuguese.

An initial lecture updated the commission about the evidence supporting each of the 2010 global strategy domains, indicating which were the most cost-effective and why. After the lecture, members of the commission responded to 57 questions in the alcohol policy scoring tool.

Each scoring domain was presented and discussed among participants and most items generated unanimous responses. Controversial items were discussed in depth and final decisions on the score were adopted by consensus. In applying the alcohol policy scoring tool, members of the commission identified the chapters and articles to be included in the new national bill. This part took 4 hours.

The bill was written in the following days. Each proposed article was compared with similar current laws and evaluated against its potential social acceptability. This writing process took 12 hours.

The alcohol policy scoring tool was used to assess Sao Tome and Principe’s existing national alcohol control laws and they scored 80 out of 494 (Table 1). Only policies 2.1, 4.1, 4.4, 5.1, 5.6, 10.1 and 10.2 (Table 2) contributed to the score. Domains 1, 3, 6, 7, 8 and 9 were not covered in the existing legislation. Thus, the existing legislation had an adequacy score of 16.2% (80/494) adequacy or a gap score of 83.8% (414/494; Table 1). In addition, the assessment revealed that the regulations were not properly enforced and/or had fallen into disuse.

**Table 1:** Alcohol policy score applied to the existing alcohol control laws, Sao Tome and Principe, 2021

Domain	Expected^a^	Actual^b^	Difference	Gap score (%)
1. Leadership, awareness and commitment	23	0	23	100.0
2. Health service response	44	15	29	65.9
3. Community and workplace action	22	0	22	100.0
4. Drink–driving policies and countermeasures	66	23	43	65.2
5. Availability of alcohol	94	21	73	77.7
6. Marketing of alcoholic beverages	48	0	48	100.0
7. Pricing policies	70	0	70	100.0
8. Reducing the negative consequences of alcohol	16	0	16	100.0
9. Reducing illicit alcohol and informal production	30	0	30	100.0
10. Monitoring and surveillance	81	21	60	74.1
**Total**	**494**	**80**	**414**	**83.8**

^a^ The total score expected by the alcohol policy score tool.^15^

^b^ Score calculated from Sao Tome and Principe actual laws and policies.

**Table 2:** Policies included in the national alcohol bill based on the alcohol policy score tool, Sao Tome and Principe, 2021

Domain, policy^a^	Included in the bill
**1. Leadership, awareness and commitment**	
1.1 National policy document on alcohol	Yes
1.2 Definition of an alcoholic beverage	Yes
1.3 Definition of a standard drink	Yes
1.4 Awareness activities	Yes
**2. Health service response**	
2.1 Screening and brief interventions for harmful/hazardous use	Yes
2.2 Special treatment programmes	Yes
2.3 Pharmacological treatment	Yes
**3. Community and workplace action**	
3.1 School-based prevention and reduction of alcohol-related harm	Yes
3.2. Workplace-based prevention of and counselling for alcohol harmful use	Yes
3.3 Community-based interventions to reduce alcohol-related harm	Yes
**4. Drink–driving policies and countermeasures**	
4.1 Maximum legal blood alcohol content limit when driving	Yes
4.2 Enforcement using sobriety checkpoints	Yes
4.3 Enforcement using random breath-testing	Yes
4.4 Penalties	Yes
**5. Availability of alcohol**	
5.1 Lowest age limit for alcohol sales and consumption	Yes
5.2 Control of retail sales	Yes
5.3 Restrictions on availability by time	No
5.4 Restrictions on availability by place	Yes
5.5 Restrictions on sales at specific events	Yes
5.6 Alcohol-free public environments	Yes
**6. Marketing of alcoholic beverages**	
6.1 Legally binding restrictions on alcohol advertising	Yes
6.2 Legally binding restrictions on product placement	Yes
6.3 Legally binding restrictions on industry sponsorship for sporting and youth events	Yes
6.4 Legally binding restrictions on sales promotions by producers, retailers and bar owners	Yes
**7. Pricing policies**	
7.1 Adjustment of taxation level for inflation	Yes
7.2 Affordability of alcoholic beverages	Yes
7.3 Other pricing measures	Partially
**8. Reducing the negative consequences of alcohol**	
8.1 Server training	No
8.2 Health-warning labels	Yes
**9. Reducing illicit alcohol and informal production**	
9.1 Use of duty-paid or excise stamps on containers	No
9.2 Estimates of unrecorded alcohol consumption	Yes
9.3 Legislation to prevent illegal production and sale of alcohol	Yes
**10. Monitoring and surveillance**	
10.1 National monitoring system	Yes
10.2 National surveys	Yes

^a^ Items in the alcohol policy score tool.^15^

Policies included in the new alcohol bill are presented in Table 2. The gaps that the new bill was expected to address were defined using the alcohol policy scoring tool. The new bill used the Cabo Verde alcohol law as a reference and incorporated the following features: the definition of a standard drink; treatment for alcohol disorders; licence for points of sale and additional restrictions by locations; prohibition of alcohol industry funding and advertising at sports and youth events; restrictions on promotions including bulk purchase models; adjustment of taxation level to inflation; and strengthening of surveillance of alcohol consumption in the population.

The new national alcohol bill has 12 chapters and 117 articles (Table 3). The bill scored 91.5% on the alcohol policy scoring tool (452 points out of 494). Only four items of the tool were not covered, namely: restrictions on hours of sale (5.3 availability); an additional tax (7.3 pricing); server training (8.1 consequences); and excise stamps (9.1 illicit alcohol). These gaps were compensated for by recommendations on advertising, promotion, treatment, prevention and epidemiological surveillance. The bill also sought to restrict interference from the alcohol industry.

**Table 3:** Structure and topics of the national alcohol control bill, Sao Tome and Principe, 2021

Structure	Topic	Domains^a,b^
**Chapter I**	**General provisions**	1
Section I	Object and scope of the bill	1
Section II	National alcohol policy and strategy	1
**Chapter II**	**Prevention of alcohol consumption**	1; 3
Section I	General provisions	1; 3
Section II	General provisions (continuation)	1; 3
**Chapter III**	**Availability of alcoholic beverages**	5; 8
Section I	General provisions	5; 8
Section II	Availability of alcoholic beverages in public and private services	5; 8
**Chapter IV**	**Import of alcoholic beverages**	5; 8; 9
**Chapter V**	**Sale of alcoholic beverages**	5; 7; 8
Section I	Wholesale sales	5; 8
Section II	Retail sales	5; 8
Section III	Prices of alcoholic beverages	7
**Chapter VI**	**Production of alcoholic beverages**	5; 8; 9
Section I	General provisions	5
Section II	Industrial production of alcoholic beverages	5; 9
Section III	Traditional producers	5; 9
Section IV	Small producers of alcoholic beverages	5; 9
**Chapter VII**	**Advertising of alcoholic beverages**	6
Section I	Marketing and advertising of alcoholic beverages	6
**Chapter VIII**	**Driving under the effect of alcoholic beverages**	4
**Chapter IX**	**Diagnosis and treatment**	2
Section I	Diagnostic systems and procedures	2
Section II	Treatment	2
**Chapter X**	**Monitoring and surveillance**	10
**Chapter XI**	**Inspection and sanctioning system**	NA
Section I	General provisions	NA
Section II	Type of offences	NA
Section III	Sanctions	NA
**Chapter XII**	**Final and transitory provisions**	NA
**Annexes**	Table of standard drink; templates for licensing and registration; list of alcoholic beverages subject to minimum unit price; and template for production registry	1; 5; 7; 9

NA: not applied.

^a^ In the alcohol policy score tool.^15^

^b^ We categorized some domains as NA since they are not included as part of the alcohol policy score even though mandatory in any law.

The new national alcohol bill emphasizes the implementation of high-impact interventions, especially those related to the three best-buys or most cost-effective population-wide interventions: increased excise taxes on alcohol; bans or comprehensive restrictions on exposure to alcohol advertising; and restrictions on the physical availability of retailed alcohol through reduced hours of sale.^26^ Usually, legislating to adopt the best-buys interventions raises considerable resistance. For example, reducing the affordability of alcoholic beverages is attacked by the alcohol industry because it affects their profit^21^ and by the population because they pay more for alcoholic products.

### Aligning the framework

The commission defined principles and complemented the bill with issues related to enforcement and sanctions. Regulatory features to facilitate the implementation process and standards proposed, such as licensing forms and measures of alcohol dose (grams of ethanol per millilitre), were inserted into the bill. Possible overlaps with existing laws were identified to ensure alignment. Due to the newness of most of the areas regulated, the bill proposed to repeal several existing laws.

### Presenting to parliament

In November 2021, the bill was sent in its final version to the National Assembly. In February 2022, the bill was upheld at the first statutory voting, despite the abstention of one of the three parties. Following this approval in the plenary session of the National Assembly, the bill was submitted to the First Commission on Political, Legal, Constitutional and Ethical Affairs. The First Commission is currently reviewing each article of the bill. The bill will then return to the National Assembly plenary session for discussion and approval. The outcome of this process will be the final version of the bill that will be then submitted to the President for its promulgation or veto. If the President vetoes the bill, it will return to parliament for review.

### Challenges

Although interference from the alcohol industry is unlikely to occur due to the small market potential in Sao Tome and Principe, the upcoming parliamentary elections may affect the outcome. The lack of a deadline for the evaluation by the First Commission, the lack of a scheduled agenda item for the plenary discussion and the short time left to the end of the current legislative mandate may result in the bill being held in the National Assembly for an indefinite period. For the bill to be tabled for plenary discussion, several things are needed: alcohol control remains a political priority; civil society organizations and other stakeholders advocate for keeping the momentum; the government provides evidence of the burden and harm caused by alcohol consumption; and international organizations raise awareness and hold the country accountable to international commitments.

### Opportunities

In its current format, the new bill would be a comprehensive reference regulation for alcohol control in Africa’s low- and middle-income countries. The process described here provides a content reference framework and a stepwise approach for other countries. After the approval of the action plan to effectively implement the global strategy to reduce the harmful use of alcohol as a public health priority, other countries will likely embark on similar initiatives.^12^^,^^13^ A different, still successful approach was followed by Malawi.^27^

## Lessons learnt

### Needs-driven, evidence-based approach

The initiative took place because a group of legislators was concerned with the harm caused by alcohol consumption. The initiative was based on, and driven by, concrete needs. This need kept up interest in continuing the process and ensured the use of evidence to facilitate discussions and reach agreements about the directions to follow.

### Inclusive and systematic process

The process for developing the bill was participatory and included the perspectives of a broad range of stakeholders (representativeness), legislators (political will and leadership) and senior experts (evidence and scientific knowledge). International standards and tools were used to facilitate the convergence towards a comprehensive bill.

### Political will for a common goal

The political will to tackle a public health problem prevailed over party interests. Agreement was facilitated by making the well-being of vulnerable communities the main goal. For example, the traditional and homemade production of alcoholic beverages was excluded from the bill due to a lack of financial resources to help those who would be affected if this aspect had been included in the law.

### Accountability of parties

Some features of Sao Tome and Principe may have facilitated the approach and process. The country is small in size and population. These features eased accountability between legislators and community members and protected the bill’s development from vested interests, especially from the alcohol industry.

### Timeliness and opportunity

The participatory process to develop the bill kept the momentum to ensure a shared vision of the interventions needed to address the problems caused by the consumption of alcohol. However, the opportunity to sustain these learning efforts may be hindered by the parliamentary procedures and timing, leading to uncertainty about the outcome.

## Conclusion

Developing a comprehensive bill for alcohol control aligned to the 10 domains of the 2010 global strategy for reducing the harmful use of alcohol using a systematic approach is an important milestone for Sao Tome and Principe. Despite the uncertainties about the outcome of the bill’s approval, the process for developing the bill has been inclusive, culturally sensitive, evidence-based, free of industry interference and country-led. This process shows that public policies that are developed through science, political commitment and broad consultation facilitate agreement towards a public health agenda.
